# Gestational age and newborn size according to parental social mobility: an intergenerational cohort study

**DOI:** 10.1136/jech-2014-205377

**Published:** 2015-06-24

**Authors:** Denise P Gigante, Bernardo L Horta, Alicia Matijasevich, Christian Loret de Mola, Aluisio J D Barros, Ina S Santos, Fernando C Barros, Cesar G Victora

**Affiliations:** 1 Post-Graduate Programme in Epidemiology, Federal University of Pelotas, Pelotas, Rio Grande do Sul, Brazil; 2 Preventive Medicine Department, Sao Paulo University; 3 Post-Graduate Programme in Health and Behaviour, Catholic University of Pelotas, Pelotas, Rio Grande do Sul, Brazil

**Keywords:** Cohort studies, Social and life-course epidemiology, Lifecourse / Childhood Circumstances

## Abstract

**Background:**

We examined the associations between socioeconomic trajectories from birth to adulthood and gestational age and birth size in the next generation, using linked data from two population-based birth cohorts carried out in a Brazilian city. By comparing socioeconomic trajectories of mothers and fathers, we attempted to identify-specific effects of maternal and paternal socioeconomic trajectory on offspring birth weight, birth length, head circumference and gestational age at birth.

**Methods:**

2 population-based birth cohort studies were carried out in 1982 and 2004 in Pelotas (Brazil); 156 mothers and 110 fathers from the earlier cohort had children in 2004. Gestational age and birth length, weight and head circumference were measured. Analyses were carried out separately for mothers and fathers. Mediation analyses assessed the role of birth weight and adult body mass index (BMI).

**Results:**

Among mothers, but not for fathers, childhood poverty was strongly associated with smaller size in the next generation (about 400 g in weight and 1.5 cm in height) and shorter gestations (about 2 weeks). Adult poverty did not play a role. For mothers, the associations with gestational age, birth length and weight—but not with head circumference—persisted after adjusting for maternal birth weight and for the height and weight of the grandmother. Maternal birth weight did not mediate the observed associations, but high maternal BMI in adulthood was partly responsible for the association with gestational age.

**Conclusions:**

Strong effects of early poverty on gestational age and birth size in the next generation were observed among mothers, but not among fathers. These findings suggest a specific maternal effect of socioeconomic trajectory, and in particular of early poverty on offspring size and duration of pregnancy.

## Introduction

Social deprivation in early life is associated with child survival, human capital and adult health.^1^ ^2^ Early exposure to poverty also affects the health and nutrition of the next generation.^3–6^

Availability of data from cohort studies, mainly restricted to populations living in high-income countries, has also the investigation of the effects of socioeconomic trajectories—from birth to adulthood—on adult health.^7^ ^8^ Some evidence from middle-income settings is also available.^9^ ^10^ Regarding studies on socioeconomic trajectories and offspring birth weight, two studies from the USA are available that describe trajectories from adolescence to adult life,^4^ ^11^ but not from early childhood. A three generation study reported positive correlations between birth weights of grandparents, parents and offspring.^5^

However, we were unable to find any reports on socioeconomic trajectories from birth to adulthood on gestational outcomes in the next generation. Since socioeconomic inequalities are associated with preterm birth and newborn size,^6^ ^12^ ^13^ such studies are justified in understanding if the timing of parental exposure to poverty is an important dimension for next generation outcomes. It is also important to investigate whether part of the intergenerational poverty effect may be mediated through poor nutrition during pregnancy and in the first years of life.^2^

Owing to the possibility of confounding, a comparison of the effects of maternal and paternal socioeconomic trajectories may help assess causality. Specific associations with maternal poverty, which are not also found for paternal poverty, would suggest a causal effect that operates through nutrition or other maternal variables. Data from the 1982 Pelotas (Brazil) birth cohort have shown that maternal birth weight and weight gain during infancy were associated with offspring birth weight, 14 15 but no association was observed between paternal and offspring birth weight. Paternal anthropometric variables and weight gain during childhood were also unrelated to offspring birth weight.^14^ Other offspring anthropometric variables were not available in this previous analysis because information was obtained through recall by the parents. A new birth cohort study was carried out in the city of Pelotas in 2004, which made it possible for us to investigate the role of early-life parental conditions on directly measured characteristics of children born to parents who belonged to the original 1982 cohort.

The main objective of this study is to examine the effect of the socioeconomic trajectories from birth to adulthood on gestational age and newborn anthropometry in the following generation, using linked data from two population-based birth cohorts carried out in the same Brazilian city. By comparing socioeconomic trajectories of mothers and fathers, we attempted to identify specific effects of maternal trajectories—with emphasis on early maternal poverty—and compare these to paternal trajectories. We also investigated whether offspring outcomes were mediated by parental birth weight and by adult body mass index (BMI), both of which may be affected by socioeconomic position, and in turn may affect offspring birth weight.

## Methods

In Pelotas, a city located in the south of Brazil, three birth cohort studies are under way. All maternities of the city were visited on a daily basis from 1 January to 31 December 1982, 1993 and 2004 and live births whose mothers lived in the urban area of Pelotas were included in the perinatal studies, which gave origin to the cohorts. Since a number of 1982 cohort individuals were expected to have children in 2004, we linked the two databases using information on names and dates of birth of the 2004 parents. Two hundred and fifty-six children born in 2004 were thus linked to mothers (n=156) and fathers (n=110) from the 1982 cohort; 10 children were born to parents who were both in the cohort. The response rate for initial recruitment was 99.3% in both the 1982 and 2004 cohorts. Further information on these two cohorts is available.^16^ ^17^

The main socioeconomic variable collected in 1982 was family income expressed in minimum wages; this information was collected as a categorical variable with five groups (≤1; 1.1–3; 3.1–6; 6.1–10; >10). The corresponding proportions of the sample in each category were 21.9%; 47.4%; 18.5%; 6.5% and 5.7%. Unfortunately, information on income as a continuous variable was not collected. Owing to the unequal numbers of individuals in each category, it was desirable to classify individuals into tertiles to allow the study of change in income levels since childhood. A principal components analysis was carried out using four variables—delivery payment mode (out-of-pocket, public free or private health insurance) and mother's schooling, height and skin colour, all of which were strongly related to socioeconomic position. The first component was used to derive a score that was then used to rank individuals within family income groups. Cut-off points were then identified within each category so that three nearly equal-sized groups were formed. To build the tertile-equivalent groups, the 1288 individuals in the lowest family income category were added to the 675 poorest individuals in the second category. The next 1979 individuals in this second category formed the second tertile, while all the remaining individuals formed the last tertile. The three groups did not have exactly the same number of individuals owing to ties in the derived score.^9^

In the 2004–2005 follow-up, total family income was collected as a continuous variable, also recoded in tertiles. Tertiles were calculated for the whole cohorts, not only for those who had children in 2004. A new variable was created—socioeconomic trajectory—by combining these two. The nine possible combinations of tertiles in 1982 and 2004 were recoded into four groups: always poor (poorest tertile in 1982 and 2004); never poor (second or third tertile on both occasions); poor→non-poor (lowest tertile in 1982 and second or third tertile in 2004); and non-poor→poor (second or third tertile in 1982 and poorest tertile in 2004).^9^

Newborns from the 2004 birth cohort were weighed by maternity staff at the time of birth using paediatric scales with a precision of 10 g, regularly calibrated on a weekly basis by cohort study staff using standard weights. Length and head circumference at birth were obtained by the interviewers trained for participation in the study using standardised procedure, within 24 h of delivery. An infantometer and an inelastic tape, both with a 1 mm precision, were used to measure length and head circumference, respectively.

Gestational age was estimated according to the following order of preference: (A) the date of the last menstrual period (either recorded on the pregnancy card or self-referred, in this order of priority); (B) ultrasonographic evaluation performed before week 20 of pregnancy; or (C) physical examination using the Dubowitz method.^18^ Gestational age was unavailable or implausible in only 13 of all newborns recruited to the cohort.

Socioeconomic variables (schooling and income) are described for members of the 1982 birth cohort who were mothers/fathers of children who belonged to the 2004 birth cohort. Anthropometric measurements and gestational age of children born in 2004 are presented by means, SD, minimum and maximum values. Means and their respective CIs were calculated for each group of social mobility and analysis of variance was used in the analyses. The effect of social mobility on anthropometric variables and gestational age on the second generation was investigated through linear regression. Adjusted analyses were conducted in three models. Anthropometric variables (weight, height, BMI and age) of the grandmother were included in the first model. The second model included all variables of the first model plus parental birth weight. Parental BMI collected in 2004 was added to model 2 and were analysed in the third model.

Regarding mediation analyses, the total direct and indirect effects were estimated using G-computation.^19^ Interactions between exposure (poverty in childhood) and mediator (maternal birth weight and maternal BMI at adulthood) were tested. There was no statistical evidence of any effect modification, but an interaction term was included in the mediation analysis anyway. The linear regression models were tested by defining residuals, examining normality and homoscedasticity.

The Medical Ethics Committee from the Federal University of Pelotas approved the cohort studies. In 1982, mothers provided oral informed consent for participation in the study. In 2004, written consent was obtained from the 1982 birth cohort members and from all mothers whose children belonged to the 2004 birth cohort.

## Results

The characteristics of the 266 mothers and fathers from the 1982 cohort who had children in 2004 are shown in table 1. In addition, the characteristics of their children at birth are shown in online supplementary table S1. Five women and five men had no information on family income in 2004, and were therefore excluded from the analyses. The mothers of the 1982 children had considerably less education than their sons and daughters. Mothers and fathers came predominantly from the poorest and middle tertiles of income in 1982, because tertiles were based on the whole sample and not only on participants who had children. In terms of tertiles in 2004, the concentration among the poor is even more marked.

**Table 1:** Socioeconomic characteristics of the mothers and fathers born in 1982 who had children in 2004

Variable	Mothers		Fathers	
	n	Per cent	n	Per cent
Maternal schooling in 1982 (years)*				
0–4	62	39.8	44	40.4
5–7	74	47.4	41	37.6
≥8	20	12.8	24	22
Own schooling in 2004–2005 (years)†				
0–4	16	10.6	8	7.6
5–7	36	23.8	34	32.4
8–10	44	29.2	36	34.3
≥11	55	36.4	27	25.7
Income in 1982 (tertiles)				
First (poorest)	70	44.9	50	45.5
Second	63	40.4	37	33.6
Third (richest)	23	14.7	23	20.9
Income in 2004–2005 (tertiles)‡				
First (poorest)	91	60.2	64	61
Second	38	25.2	25	23.8
Third (richest)	22	14.6	16	15.2
Socioeconomic trajectory‡				
Always poor	50	33.1	38	36.2
Poor→non-poor	17	11.2	9	8.6
Non-poor→poor	41	27.2	26	24.8
Never poor	43	28.5	32	30.5
Total with full information	151	100	105	100

*Schooling of the grandmother of the child born in 2004.

†Schooling of the mothers/fathers born in 1982 who had children in 2004.Variable with five missing values.

‡Variable with five missing values for women and five for men.

Those who were poor in 1982 tended to continue in the same category up to 2004. Among those who were non-poor (upper two tertiles) in 1982, almost half of those who had children had become poor by 2004.

Mean values for the perinatal outcomes among the 256 children included in the analyses are presented in table 2. All children were weighed at birth, but information on the other perinatal outcomes were missing for up to seven newborns. There were important differences for mothers but not for fathers. For mothers, early poverty, but not adult poverty, was associated with smaller size at birth in the offspring and shorter gestational age.

**Table 2:** Means and 95% CI of variables at birth in relation to socioeconomic trajectories in parents born in 1982

	Variables at birth			
	Weight (kg)	Length (cm)	Head circumference (cm)	Gestational age (week)
Mothers’ socioeconomic trajectory	p=0.001	p<0.001	p=0.012	p=0.003
Always poor	2.99 (2.83 to 3.14)	47.31 (46.59 to 48.04)	33.60 (33.12 to 34.08)	37.94 (37.11 to 38.76)
Poor→non-poor	2.91 (2.53 to 3.30)	47.26 (45.52 to 49.00)	33.57 (32.44 to 34.70)	38.06 (36.52 to 39.60)
Non-poor→poor	3.28 (3.12 to 3.43)	48.44 (47.74 to 49.14)	34.11 (33.51 to 34.72)	39.44 (38.78 to 40.10)
Never poor	3.31 (3.17 to 3.46)	49.08 (48.43 to 49.73)	34.46 (34.11 to 34.81)	39.28 (38.68 to 39.88)
Fathers’ socioeconomic trajectory	p=0.41	p=0.493	p=0.716	p=0.448
Always poor	2.99 (2.80 to 3.19)	48.05 (47.44 to 48.66)	33.74 (33.38 to 34.11)	38.32 (37.36 to 39.27)
Poor→non-poor	3.33 (3.03 to 3.63)	48.70 (47.07 to 50.33)	34.54 (33.70 to 35.38)	39.89 (38.51 to 41.27)
Non-poor→poor	3.13 (2.94 to 3.33)	48.29 (47.14 to 49.43)	33.70 (32.92 to 34.48)	38.92 (37.92 to 39.93)
Never poor	3.11 (2.89 to 3.33)	48.47 (47.79 to 49.16)	33.98 (33.57 to 34.39)	38.84 (37.95 to 39.73)

The crude and adjusted regression analyses on the effects of maternal and paternal social mobility on next generation outcomes are shown in table 3 (for birth weight and length) and in table 4 (for head circumference and gestational age). The associations between early maternal poverty with birth weight, length and gestational age persist even after adjustment for anthropometry of the grandmother, maternal birth weight and maternal BMI in 2004. The association with head circumference and maternal trajectory was in the expected direction, but it was not statistically significant. The adjusted effects of early poverty were of approximately 400 g for birth weight, 1.5 cm for birth length and 0.5 cm (non-significant) for head circumference; in addition, newborns whose mothers were never poor had a higher average gestational age of almost 2 weeks than those whose mothers were always poor.

**Table 3:** Regression coefficients and 95% CI of birth weight and birth length in relation to socioeconomic trajectory

Socioeconomic trajectory	Birth weight (kg)				Birth length (cm)			
	Crude	Model 1	Model 2	Model 3	Crude	Model 1	Model 2	Model 3
Mothers	p=0.005	p=0.001	p=0.007	p=0.003	p=0.004	p=0.009	p=0.05	p=0.04
Always poor	Reference	Reference	Reference	Reference	Reference	Reference	Reference	Reference
Poor→non-poor	−0.08 (−0.38 to 0.23)	−0.15 (−0.50 to 0.19)	−0.04 (−0.39 to 0.31)	0.01 (−0.32 to 0.35)	−0.05 (−1.47 to 1.37)	−0.48 (−2.14 to 1.17)	0.21 (−1.42 to 1.84)	0.36 (−1.26 to 1.98)
Non-poor→poor	0.29 (0.06 to 0.52)	0.5 (0.10 to 0.60)	0.35 (0.10 to 0.60)	0.38 (0.14 to 0.61)	1.13 (0.08 to 2. 17)	1.26 (0.05 to 2.46)	1.28 (0.13 to 2.44)	1.35 (0.21 to 2.49)
Never poor	0.33 (0.10 to 0.55)	0.44 (0.17 to 0.70)	0.38 (0.12 to 0.65)	0.42 (0.17 to 0.67)	1.77 (0.74 to 2.80)	1.88 (0.62 to 3.14)	1.55 (0.33 to 2.77)	1.65 (0.44 to 2.86)
Fathers	p=0.42	p=0.68	p=0.64	p=0.66	p=0.82	p=0.84	p=0.85	p=0.86
Always poor	Reference	Reference	Reference	Reference	Reference	Reference	Reference	Reference
Poor→non poor	0.33 (−0.08 to 0.76)	0.27 (−0.17 to 0.71)	0.29 (−0.16 to 0.73)	0.28 (−0.17 to 0.73)	0.65 (−1.01 to 2.31)	0.54 (−1.21 to 2.29)	0.54 (−1.23 to 2.32)	0.5 (−1.28 to 2.29)
Non-poor→poor	0.14 (−0.15 to 0.43)	0.03 (−0.32 to 0.38)	0.04 (−0.31 to 0.40)	0.04 (−0.31 to 0.40)	0.24 (−0.91 to 1.38)	−0.28 (−1.66 to 1.10)	−0.28 (−1.68 to 1.12)	−0.3 (−1.70 to 1.11)
Never poor	0.12 (−0.15 to 0.39)	0.04 (−0.29 to 0.37)	0.05 (−0.29 to 0.38)	0.05 (−0.29 to 0.38)	0.42 (−0.67 to 1.51)	0.06 (−1.37 to 1.25)	0.06 (−1.39 to 1.27)	−0.08 (−1.41 to 1.26)

Model 1: anthropometry of the grandmother (weight, height, body mass index (BMI) and age). Model 2: model 1+parental birth weight. Model 3: model 2+parental BMI in 2004.

**Table 4:** Regression coefficients and 95% CI of head circumference and gestational age in relation to socioeconomic trajectory

Socioeconomic trajectory	Head circumference (cm)				Gestational age (weeks)			
	Crude	Model 1	Model 2	Model 3	Crude	Model 1	Model 2	Model 3
Mothers	p=0.08	p=0.22	p=0.47	p=0.39	p=0.01	p=0.008	p=0.01	p=0.005
Always poor	Reference	Reference	Reference	Reference	Reference	Reference	Reference	Reference
Poor→non-poor	−0.03 (−1.00 to 0.95)	−0.28 (−1.50 to 0.95)	0.03 (−1.22 to 1.28)	0.15 (−1.09 to 1.40)	0.12 (−1.28 to 1.52)	0.54 (−1.16 to 2.24)	0.76 (−0.99 to 2.52)	0.99 (−0.72 to 2.71)
Non-poor→poor	0.52 (0.20 to 1.24)	0.43 (−0.46 to 1.33)	0.44 (−0.44 to 1.33)	0.5 (−0.38 to 1.37)	1.5 (0.45 to 2.55)	1.89 (0.65 to 3.13)	1.9 (0.66 to 3.14)	1.99 (0.79 to 3.21)
Never poor	0.86 (0.15 to 1.57)	0.87 (−0.07 to 1.80)	0.72 (−0.22 to 1.65)	0.8 (−0.13 to 1.73)	1.34 (0.30 to 2.38)	1.99 (0.69 to 3.28)	1.88 (0.57 to 3.19)	2.03 (0.75 to 3.31)
Fathers	p=0.42	p=0.44	p=0.45	p=0.47	p=0.43	p=0.43	p=0.42	p=0.39
Always poor	Reference	Reference	Reference	Reference	Reference	Reference	Reference	Reference
Poor→non-poor	0.8 (−0.25 to 1.85)	0.75 (−0.37 to 1.87)	0.75 (−0.38 to 1.89)	0.74 (−0.41 to 1.88)	1.57 (−0.40 to 3.55)	1.59 (−0.31 to 3.48)	1.63 (−0.29 to 3.55)	1.68 (−0.24 to 3.61)
Non-poor→poor	−0.04 (−0.77 to 0.68)	−0.2 (−1.08 to 0.69)	−0.19 (−1.09 to 0.71)	−0.2 (−1.10 to 0.71)	0.61 (−0.75 to 1.96)	0.31 (−1.18 to 1.80)	0.35 (−1.16 to 1.86)	0.37 (−1.15 to 1.89)
Never poor	0.23 (−0.46 to 0.92)	−0.01 (−0.85 to 0.83)	−0.01 (−0.86 to 0.85)	−0.01 (−0.87 to 0.85)	0.53 (−0.75 to 1.81)	0.35 (−1.07 to 1.77)	0.37 (−1.06 to 1.81)	0.4 (−1.04 to 1.83)

Model 1: anthropometry of the grandmother (weight, height, body mass index (BMI) and age). Model 2: model 1+parental birth weight. Model 3: model 2+parental BMI in 2004.

Table 5 shows that maternal birth weight and BMI at adulthood did not mediate the effect of poverty in childhood on offspring birth weight and birth length, as indirect effects were small. Maternal smoking captured about 13% of the effect of poverty in childhood on birth weight. For gestational age, maternal BMI at adulthood captured about one-fourth of the total effect (with a CI barely including the zero value), whereas maternal birth weight was not a mediator. The full set of estimates from mediation analysis is shown in online supplementary table S2.

**Table 5:** Estimated direct and indirect effects of poverty in childhood mediated through maternal birth weight, smoking and body mass index (BMI) in adulthood (CI 95%)

Mean difference: offspring of poor mother in childhood vs offspring of non-poor mother in childhood			
Mediator	Total effect	Direct effect	Indirect effect
Outcome: offspring birth weight in g			
Maternal birth weight	−366.5 (−587.8 to −145.1)	−375.0 (−597.9 to −152.1)	8.6 (132.7 to −115.6)
Maternal BMI in 2004	−276.7 (−507.2 to −46.1)	−328.6 (−553.8 to −103.4)	51.9 (190.4 to −86.6)
Maternal smoking in 2004	−376.6 (−594.8 to −158.4)	−328.3 (−547.0 to −109.6)	−48.3 (−163.3 to 66.8)
Outcome: offspring birth length in cm			
Maternal birth weight	−0.74 (−1.77 to 0.29)	−0.84 (−1.87 to 0.18)	0.11 (0.67 to −0.46)
Maternal BMI in 2004	−1.01 (−2.08 to 0.06)	−1.38 (−2.47 to 0.29)	0.37 (0.96 to −0.22)
Maternal smoking in 2004	−1.13 (−2.20 to −0.06)	−1.13 (−2.20 to −0.06)	0.00 (−0.55 to 0.54)
Outcome: offspring head circumference in cm			
Maternal birth weight	−0.70 (−1.44 to 0.04)	−0.45 (−1.19 to 0.29)	−0.25 (−0.64 to 0.14)
Maternal BMI in 2004	−0.73 (−1.49 to 0.04)	−0.80 (−1.58 to 0.02)	0.08 (0.49 to −0.34)
Maternal smoking in 2004	−1.06 (−1.84 to −0.27)	−1.04 (−1.82 to −0.26)	−0.02 (−0.40 to 0.36)
Outcome: offspring gestational age in weeks			
Maternal birth weight	−1.64 (−2.69 to −0.59)	−1.64 (−2.69 to 0.59)	0.00 (−0.56 to 0.56)
Maternal BMI in 2004	−2.21 (−3.27 to −1.16)	−1.60 (−2.67 to −0.54)	−0.61 (−1.20 to −0.01)
Maternal smoking in 2004	−2.08 (−3.14 to −1.03)	−2.24 (−3.30 to −1.18)	0.16 (−0.39 to 0.71)

## Discussion

The major strength of this study is the linkage of data from two prospective, population-based cohort studies. Since both cohorts included all births in the city of Pelotas in a calendar year, it was possible to identify a substantial number of births. In 2005, 75% of the 1982 cohort individuals were traced during a wave of data collection, so that the vast majority of the cohort was still resident in the city. Owing to the higher fertility and younger childbearing age, participants from the 1982 cohort who were born to low-income families were more likely to have had a child in 2004. This applied to mothers and fathers. A limitation of the present analyses, therefore, was that only births to relatively young parents (aged 21–21 years) were studied; it will be interesting to repeat the analyses when data from the ongoing 2015 Pelotas birth cohort become available.

The strengths of our study include its prospective design, population-based samples and the fact that measures were taken directly by the research team using standardised methods, rather than obtained through recall or from records.

There are several reports in the literature on the association between birth weight in successive generations.^4–5^ ^12–15^ ^20^ There are also reports on the detrimental effects of poverty at different ages on offspring birth weight.^4^ ^11^ ^12^ ^21^ The present article contributes to this literature by investigating the effects of early poverty and of lifetime socioeconomic trajectories of mothers and fathers, allowing for parental birth weight. We also report on the effects of parental socioeconomic trajectories on birth length, head circumference and gestational age, unlike previous studies that were based on birth weight alone.

The initial focus of the analyses was on socioeconomic trajectories, but our findings show that early maternal poverty has a lasting effect, regardless of whether or not the woman remained poor. Poverty in childhood, but not at an adult age, is associated with sizeable reductions in offspring birth weight, birth length, head circumference and gestational age. Gavin had previously reported a similar pattern of association with poverty school age (11–13 years), but not adulthood, and offspring birth weight. In contrast, we found no association with paternal poverty or socioeconomic trajectory. Associations between socioeconomic factors and offspring birth weight that were specific for mothers, but not for fathers, had been previously reported.^20^

The specificity of the finding for mothers, rather than fathers, suggests that this association is not due to confounding but has a biological mechanism, possibly through maternal nutrition in early life. The importance of early determinants in offspring birth weight is confirmed by other studies. Previous analyses of five cohort studies from low-income and middle-income countries, including the Pelotas cohort, show that mothers who were stunted at the age of 2 years had offspring whose birth weight was on average 80 g lighter than children born to mothers who were not stunted in early life.^2^ In a Swedish cohort, the intergenerational transmission of birth weight was partly explained by environmental factors.^5^

The reported effects for birth weight were also observed for birth length, including the specificity of maternal rather than paternal socioeconomic trajectories. Gestational age was also associated with maternal trajectories in a similar fashion, with early poverty resulting in pregnancies that were about 2 weeks shorter in the adjusted analyses, a sizeable effect. We failed to identify any earlier reports on this association. However, head circumference did not seem to be associated with poverty of the mother or of the father, possibly an indication of a brain-sparing effect in which other parts of the fetal body are sacrificed on behalf of preserving brain tissue.^22^

Our analyses suggest that maternal birth weight did not mediate the effect of early poverty on offspring birth weight and birth length, as indirect effects were small. For gestational age, maternal BMI at adulthood captured about one-fourth of the total effect. Poor women are more likely to be overweight or obese in our cohort, 23 and excessive weight is a well-known risk factor for preterm delivery.^24^ Thus, this finding has biological plausibility.

Our findings support the concept of the 1000 days window of opportunity, from conception to the age of 2 years, as essential not only to adult health and human capital^2^ but also to the nutrition of the next generation. Interventions directed at this critical window—including not only nutritional interventions but also social programmes—are likely to have long-lasting effects.

**What is already known on this subject?**

- Early exposure to poverty among mothers affects the birth size and gestational age of their offspring. Limited evidence is available for the effects of paternal poverty on health and nutrition of the next generation. The current socioeconomic position of the family is an important determinant of size at birth, and to a lesser extent of preterm delivery.

**What this study adds?**

- Early but not current maternal poverty is associated with offspring birth weight, birth length and gestational age in a prospective two general study in Brazil. These variables were not associated with paternal poverty. The effect of early poverty on birth weight and birth length was not mediated either through maternal birth weight or current body mass index (BMI). Our findings support the concept that exposure to adverse socioeconomic conditions during the first 1000 days, from conception to the age of two years, can affect the nutrition of the next generation.

## Supplementary Material

Web table 1

Web table 2
