# Supplementary material for: Gestational age and newborn size according to parental social mobility: an intergenerational cohort study
Source: J Epidemiol Community Health. 2015 Jun 24;69(10):944–9. doi: 10.1136/jech-2014-205377 (PMC4602273; doi:10.1136/jech-2014-205377)
Supplement: Web table 1 [file jech-2014-205377-s1.pdf]

Supplementary Table 1. Characteristics of the children at birth.

Variable at birth	n	Means	SD	Minimum	Maximum
Weight (g)	256	3120	588	680	4150
Length (cm)	249	48.19	2.42	37	53.1
Head circumference (cm)	249	33.96	1.61	26.5	37
Gestational age (week)	255	38.71	2.77	25	44
