# Supplementary material for: Gestational age and newborn size according to parental social mobility: an intergenerational cohort study
Source: J Epidemiol Community Health. 2015 Jun 24;69(10):944–9. doi: 10.1136/jech-2014-205377 (PMC4602273; doi:10.1136/jech-2014-205377)
Supplement: Web table 2 [file jech-2014-205377-s2.pdf]

Supplementary Table 2. Exposure: Early wealthy compared to early poor. Regression coefficient stratified by mediator (CI 95%)

	Total controlled effect	Natural direct effect	Natural indirect effect	Controlled direct effect
**Birthweight - grams**				
Maternal birthweight				
2500	202.9 (-22.0; 427.9)	277 (52.4; 501.7)	-74.1 (-196.8; 48.6)	254.6 (34.1; 475.1)
3000	366.5 (145.1; 587.8)	375 (152.1; 597.9)	-8.6 (-132.7; 115.6)	287.3 (69.5; 505.0)
3500	141.3 (-81.7; 364.4)	274.9 (49.1; 500.7)	-133.6 (-257.4; -9.8)	224.6 (6.6; 442.5)
Maternal BMI 2004				
20	276.7 (46.1; 507.2)	328.6 (103.4; 553.8)	-51.9 (-190.4; 86.6)	448.5 (225.5; 671.6)
25	298.6 (66.7; 530.4)	358.9 (133.5; 584.3)	-60.4(-198.7; 78.0)	379.8 (157.1; 602.4)
30	344.1 (114.0; 574.2)	240.6 (16.0; 465.2)	103.5 (-34.0; 241.1)	370.9 (147.5; 594.3)
Maternal smoking				
No	444.4 (219.9; 668.9)	365.1 (141.8; 588.4)	79.3 (-39.0; 197.6)	403.0 (179.4; 626.6)
Yes	376.6 (158.4; 594.8)	328.3 (109.6; 547.0)	48.3 (-66.8; 163.3)	324.5 (104.4; 544.6)
**Birth length – centimetres**				
Maternal birthweight				
2500	0.84 (-0.20; 1.88)	1.47 (0.44; 2.50)	-0.63 (-1.19; -0.07)	0.85 (-0.16; 1.86)
3000	0.74 (-0.29; 1.77)	0.84 (-0.18; 1.87)	-0.11 (-0.67; 0.46)	1.39 (0.37; 2.41)
3500	0.65 (-0.38; 1.69)	1.14 (0.11; 2.16)	-0.48 (-1.04; 0.08)	1.04 (0.02; 2.06)
Maternal BMI 2004				
20	1.01 (-0.06; 2.08)	1.38 (0.29; 2.47)	-0.37 (-0.96; 0.22)	1.32 (0.25; 2.40)
25	1.36 (0.27; 2.45)	1.26 (0.17; 2.35)	0.10 (-0.50; 0.69)	1.46 (0.38; 2.53)
30	1.28 (0.21; 2.36)	1.34 (0.26; 2.42)	-0.06 (-0.66; 0.53)	1.59 (0.51; 2.67)
Maternal smoking				
No	1.87 (0.80; 2.94)	1.71 (0.64; 2.79)	0.15 (-0.39; 0.70)	1.60 (0.52; 2.68)
Yes	1.13 (0.06; 2.20)	1.13 (0.06; 2.20)	0.00 (-0.54; 0.55)	1.64 (0.57; 2.71)

Supplementary Table 2. Continuing

	Total controlled effect	Natural direct effect	Natural indirect effect	Controlled direct effect
**Head circumference – centimetres**				
Maternal birthweight				
2500	0.76 (0.01; 1.51)	0.80 (0.05; 1.55)	-0.05 (-0.43; 0.34)	0.62 (-0.12; 1.35)
3000	0.70 (-0.04; 1.44)	0.45(-0.29; 1.19)	0.25 (-0.14; 0.64)	0.08 (-0.65; 0.81)
3500	0.59(-0.16; 1.33)	0.49 (-0.25; 1.23)	0.10 (-0.29; 0.48)	0.49 (-0.24; 1.22)
Maternal BMI 2004				
20	0.73 (-0.04; 1.49)	0.8 (0.02; 1.58)	-0.08 (-0.49; 0.34)	0.88 (0.10; 1.65)
25	0.35 (-0.41; 1.12)	0.98 (0.20; 1.76)	-0.63 (-1.04; -0.21)	0.96 (0.17; 1.74)
30	0.44 (-0.31; 1.20)	0.74 (-0.04; 1.52)	-0.30 (-0.72; 0.12)	0.50 (-0.27; 1.28)
Maternal smoking				
No	1.18 (0.39; 1.97)	1.05 (0.26; 1.84)	0.13 (-025; 0.50)	0.54 (-025; 1.32)
Yes	1.06 (0.27; 1.84)	1.04 (0.26; 1.82)	0.02 (-0.36; 0.40)	0.66 (-0.13; 1.44)
**Gestational age – weeks**				
Maternal birthweight				
2500	1.81 (0.76; 2.86)	1.86 (0.82; 2.90)	-0.05 (-0.62; 0.51)	1.19 (0.15; 2.24)
3000	1.64 (0.59; 2.69)	1.64 (0.59; 2.69)	0.00 (-0.56; 0.56)	1.56 (0.52; 2.61)
3500	1.14 (0.10; 2.18)	1.45 (0.41; 2.48)	-0.30 (-0.86; 0.25)	1.66 (0.63; 2.69)
Maternal BMI 2004				
20	2.21 (1.16; 3.27)	1.6 (0.54; 2.67)	0.61 (0.01; 1.20)	2.12 (1.07; 3.17)
25	1.52 (0.47; 2.58)	1.84 (0.79; 2.90)	-0.32 (-0.91; 0.27)	1.79 (0.75; 2.82)
30	2.29 (1.24; 3.35)	1.66 (0.59; 2.72)	0.64 (0.04; 1.23)	2.12 (1.06; 3.18)
Maternal smoking				
No	1.65 (0.60; 2.69)	1.67 (0.63; 2.71)	-0.02 (-0.57; 0.53)	2.17 (1.12; 3.22)
Yes	2.08 (1.03; 3.14)	2.24 (1.18; 3.30)	-0.16 (-0.71; 0.39)	1.79 (0.74; 2.84)
